# Mechanism of SET8 Activates the Nrf2-KEAP1-ARE Signaling Pathway to Promote the Recovery of Motor Function after Spinal Cord Injury

**DOI:** 10.1155/2023/4420592

**Published:** 2023-03-10

**Authors:** Xin Li, Yan Qian, Wanling Shen, Shiying Zhang, Hui Han, Yu Zhang, Shuangmei Liu, Shaokun Lv, Xiuying Zhang

**Affiliations:** Rehabilitation Medicine of Qujing No. 1 Hospital, Qujing, 655000 Yunnan, China

## Abstract

**Background:**

Spinal cord injury (SCI) is a common injury of the central nervous system (CNS), and astrocytes are relatively abundant glial cells in the CNS that impairs the recovery of motor function after SCI. It was confirmed that the oxidative stress of mitochondria leads to the accumulation of reactive oxygen species (ROS) in cells, which plays a key role in the motor function of astrocytes. However, the mechanism by which oxidative stress affects astrocyte motility after SCI is still unexplained. Therefore, this study investigated the influence of SET8-regulated oxidative stress on astrocyte autophagy levels after SCI in rats and the potential mechanisms of action.

**Methods:**

We used real-time quantitative PCR, western blotting, and immunohistochemical staining to analyze SET8, Keap1, and Nrf2 expression at the cellular level and in SCI tissues. ChIP to detect H4K20me1 enrichment in the Keap1 promoter region under OE-SET8 (overexpression of SET8) conditions. Western blotting was used to assess the expression of signature proteins of astrocytes, proteins associated with autophagy, proteins associated with glial scar formation, reactive oxygen species (ROS) levels in cells using DHE staining, and astrocyte number, morphological alterations, and induction of glial scar formation processes using immunofluorescence. In addition, the survival rate of neurons after SCI in rats was examined by using NiSSl staining.

**Results:**

OE-SET8 upregulates the enrichment of H4K20me1 in Keap1, inhibits Keap1 expression, activates the Nrf2-ARE signaling pathway to suppress ROS accumulation, inhibits oxidative stress-induced autophagy and glial scar formation in astrocytes, and leads to reduced neuronal loss, which promoted the recovery and improvement of motor function after SCI in rats.

**Conclusion:**

Overexpression of SET8 alleviated oxidative stress by regulating Keap1/Nrf2/ARE, inhibited astrocyte autophagy levels, and reduced glial scar formation as well as neuronal loss, thereby promoting improved recovery of motor function after SCI. Thus, the SET8/H4K20me1 regulatory function may be a promising cellular therapeutic intervention point after SCI.

## 1. Preface

Spinal cord injury (SCI) is a common central nervous system (CNS) injury that causes movement dysfunction and even paralysis. In recent years, the prevalence of SCI has been increasing and trending younger, with over two million SCI cases worldwide and an annual increase of 12 thousand new cases [1]. The incidence of SCI has been increasing in recent years, and the trend is in younger populations. Pathophysiological studies have found that SCI occur and develop in two main stages, with the first stage being the primary injury caused by physical tension. In the second stage, SCI could cause secondary injury, such as mitochondrial dysfunction, oxidative stress damage, and apoptosis [2]. Secondary injury can produce neuronal apoptosis and generate glial scars [3, 4], which further inhibit spinal cord regeneration after SCI [5]. SCI has a significant influence on the recovery of motor function. Therefore, exploring the mechanisms that inhibit or interrupt secondary injury holds promise for promoting recovery of motor function after SCI.

As a highly conserved mechanism of selective intracellular degradation, autophagy mainly occurs through the lysosomal pathway as the steady response of cells to stress stimulation [6]. While most studies on autophagy have focused on immune regulation of tumors, an increasing number of researchers have confirmed that autophagy has a key role in central nervous system (CNS) diseases, especially after SCI, in recent years [7, 8]. Astrocytes are the most abundant and widely distributed class of glial cells in the CNS and have a key role in synapse formation, the production of nutritional factors, the uptake and release of neurotransmitters, and the control of neuron survival [9]. After SCI, astrocytes show abnormal “responsiveness” in the form of upregulation of GFAP and cellular hypertrophy [10]. In addition, astrocytes migrate to the lesion and proliferate, leading to the formation of glial scars [11]. The underlying mechanism is astrocyte autophagy, and Kanno et al. [12] found that astrocyte autophagy was activated and Beclin1 expression was upregulated after SCI [13], showing that astrocyte autophagy is a potential mechanism for astrocyte activation. Activated astrocytes are closely associated with neuronal injury [14, 15]. A study showed that motor neurons cocultured with astrocytes died after 72 hours [4]. Therefore, inhibition of astrocyte autophagy may improve functional prognosis after SCI.

Reactive oxygen species (ROS) are a universal or general name for a class of single-electron reduction products of oxygen with high oxidative activity or ions in the organism, which are produced by mitochondria and act as redox signal messengers in the organism [16]. After SCI, ROS in mitochondria increase [17]. ROS are produced by the mitochondria and as redox signaling messengers in the body, leading to the formation of oxidative stress [18]. Oxidative stress directly affects the function of astrocytes [19], and induces autophagy in astrocytes [20]. Therefore, an effective way to protect the injured spinal cord from mitochondrial oxidative stress is to inhibit the production of ROS. The keap1-Nrf2/ARE pathway is the main defense mechanism of cells against oxidative stress, in which Keap1 can negatively regulate Nrf2 and the activated Nrf2 molecule can activate antioxidant genes by combining with antioxidant response elements (AREs) [21]. It was shown that activated Nrf2 prevents astrocytes from killing cocultured motor neurons in a glutathione-dependent mechanism [22, 23]. Thus, the Keap1-Nrf2/ARE signaling pathway could reduce or inhibit astrocytes autophagy and motor neuron apoptosis under the activated state, promoting a potential therapeutic approach for motor function recovery after SCI.

SET domain-containing protein 8 (SET8) is a lysine monomethyltransferase, also called SETD8, which specifically methylates H4 lysine 20 [24]. SET8/H4K20me1 participates in the regulation of many signaling pathways, including the induction of apoptosis in cancer cells [25], mediating endothelial cell inflammation [26] and cellular oxidation [27]. Chen et al. [28] showed that SET8/H4K20me1 regulates Keap1-Nrf2/ARE to inhibit ROS accumulation from oxidative damage in hyperglycemia-induced endothelial injury. This study hypothesized that SET8/H4K20me1 plays a key role in the regulation of the Keap1-Nrf2/ARE signaling pathway after SCI, thereby participating in the regulation of autophagy in astrocytes and promoting the recovery of motor function after SCI.

## 2. Method

### 2.1. Laboratory Animals

Healthy female SD rats of the SPF class (Animal Experiment Center of Kunming Medical University), weighing 275 ± 25 g, were utilized. The experimental scheme of this study was approved by the Animal Ethics Committee of Hunan Provincial People's Hospital, and it fully met the requirements of the National Institutes of Health Laboratory Animal Care Guide. This study included the following groups: sham-operated group (*n* = 10), SCI+OE-sET8 group (*n* = 10), and SCI group (*n* = 20), all the animals were distributed randomly.

### 2.2. Post-SCI Model Group in Rats

Before the skin and muscles on the covering layer were cut, rats were anesthetized with 4% pentobarbital sodium, and a laminectomy was performed on T9-11 to keep the dura mater intact. Epidural impingement was performed on the rat spinal cord with a weight-reducing impinger (20 g) placed into the T10 region of the spinal cord after descending 0.25 cm [29]. After the operation, to prevent dehydration, all animals were injected with 0.9% saline (30 mL/kg). In this study, all rats were housed in cages with controlled feeding conditions individually, with enough food and water, and had a light/dark cycle at 12-hour intervals. Assisted urination was performed 3 times daily after SCI during the study period.

### 2.3. Astrocyte Culture

Rat astrocytes were provided by Wuhan Pronosai Life Co. Rat astrocytes were inoculated at 1 × 10^5^ cells/mL and cultured at 37°C with saturated humidity and CO_2_. Before the cells covered 90% of the bottom of the culture dish, the medium was changed every other day. To purify the astrocytes, the mixed astrocytes were placed at a concentration of 1 × 10^5^ cells/mL into culture dishes coated with polylysine. Ara-C medium (5 *μ*g/mL) was added after 24 h of culture and replaced with DMEM+10% fetal bovine serum after 48 h of culture. During 10 days of continuous cell culture, the medium was changed every 3 days.

### 2.4. Chromatin Immunoprecipitation Analysis

A ChIP Assay Kit (Beyotime, Shanghai, China) was used to perform the ChIP assays in this study. AsTs (1 × 10^7^ cells) were fixed with 1% formaldehyde for 10 min to crosslink DNA and protein, and the crosslinking reaction was stopped by adding glycine. Subsequently, the cells were ultrasonically treated with a Microson ultrasonic cell disruptor to shear chromatin (every 15 s, every 2 min, power = 15 W, and amplitude = 10). Ten microliters of the ultrasonically treated extract was obtained from each group as an input sample, and the anti-h4k20me1 antibody or IgG negative control (Abcam, Cambridge, UK) was added to the remaining samples and incubated at 4°C for 12 h. DNA–protein crosslinks were reversed with protein G magnetic beads bound to the immunoprecipitates for 2 h at 65°C. Subsequently, the enriched DNA fragments were purified and analyzed by qPCR. The primers for Keap1 were as follows: 5′-TGACAAAACTGAGCCTCCTAGC-3′ and Rev 5′-GCATCAAAGAGTGATGCTGAATG-3′.

### 2.5. ROS Detection

ROS was tested by the ROS Assay Kit (Beyotime, Shanghai, China). In this study, rats were euthanized 3 days after SCI. Samples were lysed using 0.01 mol/L PBS to suspend the brain tissue and centrifuged at 500 × g for 10 min at low temperature (4°C). Then, the samples were mixed with 190 *μ*L supernatant and 10 *μ*L dichlorodihydrofluorescein diacetate (DCFH-DA; 1 M) in microtiter wells for 30 minutes at room temperature. Then, a BCA Protein Assay Kit (Beyotime, Shanghai, China) was used to obtain the protein levels. Ultimately, the ROS levels were displayed as fluorescence/mg protein. Similarly, the mixture of astrocytes and 10 micron DCFH-DA was incubated in a 96-well plate at 25°C for 30 minutes and measured using a fluorometer.

### 2.6. NiSSL Dyeing

After drying, 7 micron transverse frozen slices were directly immersed in the mixture (ethanol/chloroform = 1 : 1) for 12 h in the dark at 22 ± 1°C. Then, the sections were dyed with 0.1% cresyl violet (Sango Biotech, Shanghai, China) solution for 5 min, divided, dehydrated, and rinsed. Finally, the slices were fixed using Permount™ and observed using an Olympus optical microscope (Leica DMI4000B, Germany).

### 2.7. Quantitative Real-Time Polymerase Chain Reaction (qRT–PCR)

In this study, we extracted total RNA from tissues and cells using a Total RNA Extractor (Sangon Biotech). A cDNA synthesis kit (Vazyme, Nanjing, China) was used to reverse transcribe 2 *μ*g mRNA into cDNA, which was then diluted 10 times. One microliter of the prepared cDNA was used for qPCR. All primers (Table 1) used in this study were designed with Premier 5.0. The two-step reaction conditions for PCR were as follows: predenaturation (maintained at 95°C for 5 min), maintained at 95°C for 10 s, annealing (30 s), and extension (30 s). Both annealing and extension were cycled 40 times. The confidence of the PCR results was assessed by the dissociation curve and cycle threshold (CT) values. The results were calculated by the 2^-*ΔΔ*Ct^ method after repeated at least 3 times.

### 2.8. Western Blotting

In this study, proteins (including nuclear and cytoplasmic proteins) in spinal cord-injured rats were extracted by using RIPA lysis buffer (Sangon Biotech, Shanghai), and the total protein concentration was determined using a BCA assay (Sangon Biotech, Shanghai). A 10% SDS–PAGE gel was used to separate the total proteins, which were then transferred to PVDF membranes by constant current flow at 200 mA. Subsequently, PVDF membranes were cultured with antibodies (Abcam, USA) for 12 h at 4°C. The PVDF membranes were washed with TBS buffer and incubated with secondary antibodies (Abeam) at 25°C for 1 h. After washing the membranes 3 times, chemiluminescent reagents were added, and the bands were analyzed for grayscale values using ImageJ software. Each experiment was repeated 3 times independently.

### 2.9. Immunohistochemistry (IHC)

In this study, IHC experiments were carried out by 3,3′-diaminobenzidine (DAB) analysis. After the glass slide was baked at 65°C for 2 h, it was placed in xylene for 10 minutes. The sections were incubated in the following ethanol gradient (5 min for each solution): 90%, 80%, 70%, and distilled water. In a wet room, citric acid buffer was used to treat the slices, and hydrogen peroxide (3%) was used to remove endogenous peroxidase (25°C, 10 min). Sections were blocked with 5% bovine serum at 37°C for 30 min and then incubated with anti-Set8 and anti-keap1 antibodies (1 : 200) for 12 h at 4°C. They were incubated with goat anti-rabbit antibodies (IgG, 1 : 100) for 30 min at 37°C after washing the slices with PBS buffer. 3,3′-DAB was used to observe the sections, and a light microscope was used to acquire the images.

### 2.10. Immunofluorescence

On the 3rd, 7th, and 21st days after SCI, all rats were euthanized with deep pentobarbital and then infused with 0.1 mol/L PBS and 4% paraformaldehyde (PFA). AsTs of each group were resuspended and inoculated onto sterile coverslips coated with polylysine and then dissolved in goat serum containing 0.3% Triton X-100. Subsequently, the cells were incubated with 5% BSA for 1 h, and primary antibody (1 : 200, Abcam, UK) was added and incubated at 4°C for 12 h. Afterward, the AsTs were incubated with the corresponding fluorescent secondary antibodies. The nuclei were stained with DAPI, and images were obtained by fluorescence microscopy.

## 3. Results

### 3.1. Low Expression of Keap1 Alleviates LPS-Induced Oxidative Damage Induction and Inhibits Astrocyte Autophagy

**Figure 1:**
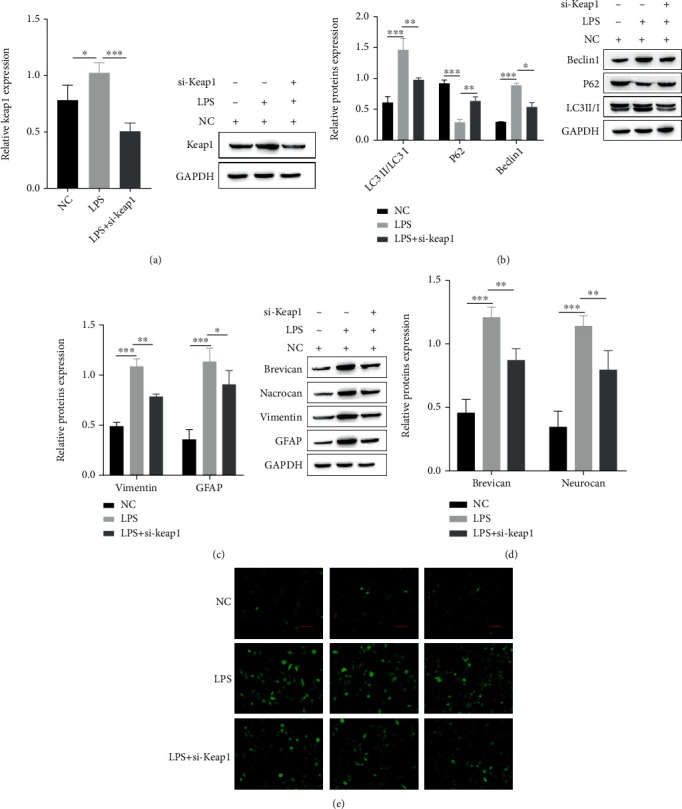
si-Keap1 alleviates LPS-induced oxidative damage induction and inhibits astrocyte autophagy. (a) Protein expression of Keap1 in astrocytes under LPS conditions under normal, LPS, and Keap1 knockdown conditions. (b) Expression of the associated autophagy proteins LC3II/I, P62, and Beclin1 in astrocytes under NC, LPS and LPS+Keap1 conditions. (c) Expression of the signature proteins GFAP and Vimentin of astrocytes under NC, LPS, and LPS+Keap1 conditions. (d) Expression of brevican and neurocan, a protein associated with glial scar formation, in NC, LPS, and LPS+Keap1 conditions. (e) DHE staining to detect the level of ROS in astrocytes under NC, LPS, and LPS+Keap1 conditions.

After SCI, mitochondrial dysfunction leads to oxidative stress in rats; astrocytes migrate to the lesion site and become abnormally “reactive,” preventing recovery of motor function and leading to glial scar formation after SCI. KEAP1 is a sensor of the oxidative stress pathway [30]. To detect the potential influence of KEAP1 on oxidative stress behavior after SCI, we used LPS to induce oxidative stress behavior in simulated rats after SCI and explored the effect of KEAP1 knockdown on LPS-induced astrocyte autophagy after oxidative injury. The experimental data showed that LPS enhanced the accumulation of ROS (Figure 1(e)) and glial scar formation (Figure 1(d)), activated astrocytes and enhanced astrocyte autophagy, as evidenced by enhanced expression of GFAP and Vimentin (Figure 1(c)) and autophagy-related proteins LC3II/I and Beclin1, while P62 expression was diminished (Figure 1(b)). In addition, knockdown of Keap1 downregulated Keap1 expression compared to the LPS (Figure 1(a)), which alleviated the oxidative stress response under LPS induction, as evidenced by a significant decrease in ROS accumulation detected by DHE staining (Figure 1(e)). It also impaired astrocyte autophagy as well as glial scar formation, and immunoblotting revealed diminished expression of the astrocyte autophagy-related proteins LC3II/I and Beclin1, while P62 expression was enhanced (Figure 1(b)). These results suggest that si-KEAP1 alleviates LPS-induced oxidative damage and attenuates astrocyte autophagy and glial scar formation after LPS.

### 3.2. Low Expression of Keap1 Reduces the Inhibitory Effect on the Nrf2-ARE Signaling Pathway

**Figure 2:**
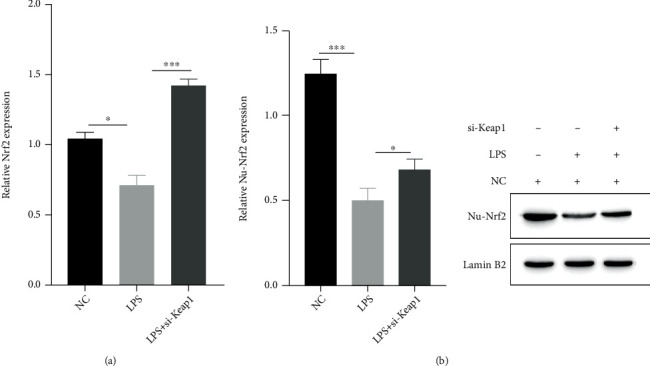
si-Keap1 reduces the inhibitory effect on the Nrf2-ARE signaling pathway. (a) RT–qPCR analysis of Nrf2 mRNA expression under NC, LPS, and LPS+Keap1 conditions; (b) expression of the signaling pathway protein Nrf2 under NC, LPS, and LPS+Keap1 conditions.

To detect the influence of KEAP1 on the Nrf2/ARE signaling pathway after SCI in rats, we used transfection of si-Keap1 to induce LPS-treated astrocytes. The effect of si-Keap1 was verified by qPCR and western blotting analysis. si-Keap1 reversed the LPS-mediated inhibition of the Nrf2/ARE signaling pathway (Figures 2(a) and 2(b)). Keap1 negatively regulates the Nrf2/ARE signaling pathway in oxidative injury in rat SCI.

### 3.3. SET8 Reduces Keap1 Expression by Enhancing H4K20me1 Enrichment in the Keap1 Promoter Region

**Figure 3:**
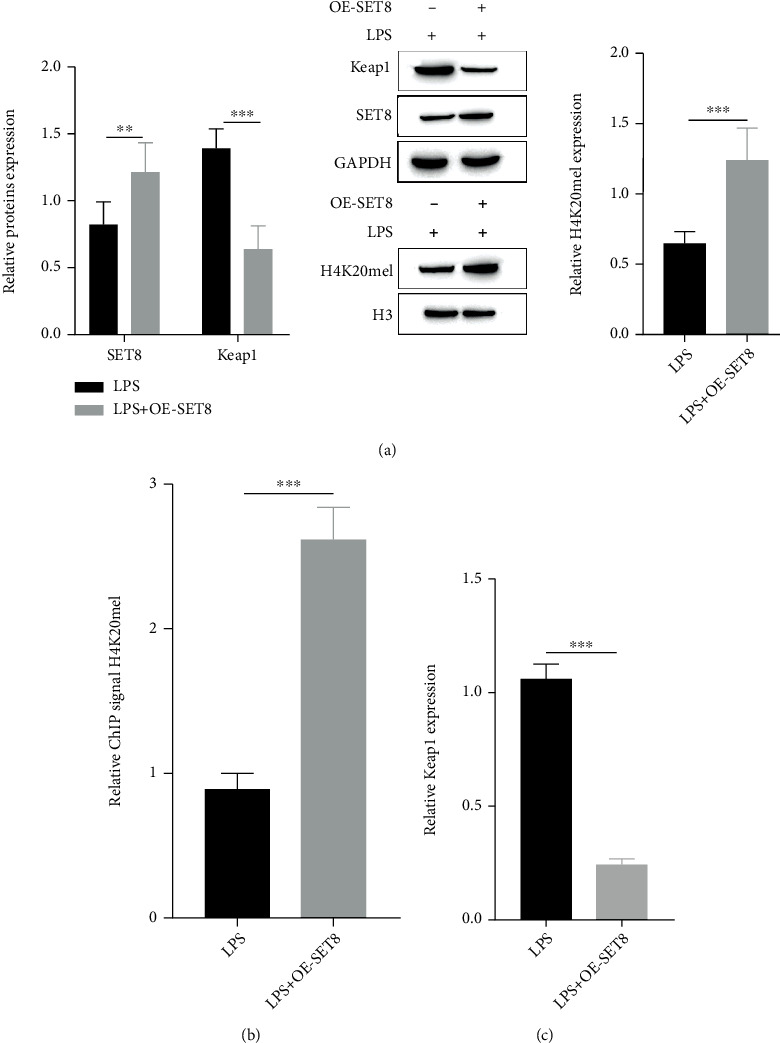
SET8 reduces Keap1 expression by enhancing H4K20me1 enrichment in the Keap1 promoter region. (a) Western blotting analysis of Keap1 and H4K20me1 expression under different conditions of NC+LPS and NC+LPS+OE-SET8; (b) ChIP detection of H4K20me1 enrichment in the Keap1 promoter region; (c) RT–qPCR analysis of Keap1 mRNA expression under different conditions of NC+LPS and NC+LPS+OE-SET8 expression under different conditions.

SET8 is a key regulator of DNA methylation and is the only known modifier enzyme that catalyzes monomethylation of histone H4K20me1 [31]. We investigated whether SET8 regulates the expression of Keap1, a key factor in oxidative stress. First, we used protein blotting and RT–qPCR to detect H4K20me1 levels in OE-SET8 cells. The results showed that H4K20me1 levels were significantly upregulated in OE-SET8 cells (Figure 3(a)). Second, this study tested the distribution of H4K20me1 in astrocyte genomes by the ChIP method, and the results showed that H4K20me1 can be enriched in the Keap1 promoter (Figure 3(b)). In addition, compared with the LPS, OE-SET8 significantly decreased Keap1 mRNA expression, as shown by protein blotting and qPCR (Figures 3(a) and 3(c)). This result indicated that SET8 inhibits Keap1 by promoting the enrichment of H4K20me1 in the Keap1 promoter, which in turn activates the Nrf2-ARE signaling pathway.

### 3.4. SET8 Inhibits Oxidative Stress-Induced Autophagy and Glial Scar Formation in Astrocytes through the KEAP1-Nrf2-ARE Signaling Pathway

**Figure 4:**
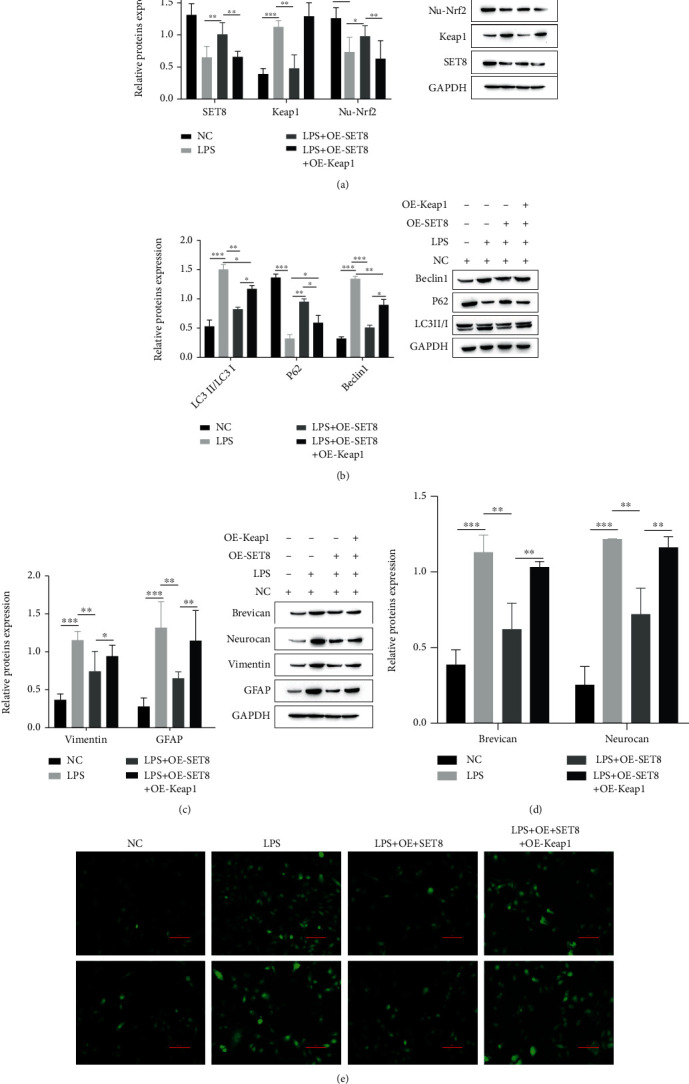
SET8 inhibits oxidative stress-induced autophagy and glial scar formation in astrocytes via the KEAP1-Nrf2-ARE signaling pathway. (a) Western blotting analysis of the signaling pathway-related proteins Keap1 and Nrf2; (b) the astrocyte signature proteins GFAP and Vimentin; (c) the astrocyte autophagy-related proteins LC3II/I, P62, and Beclin1; (d) the glial scar formation-related proteins brevican and neurocan in the NC+ LPS, NC+LPS+OE-SET8, and NC+LPS+OE-SET8+OE-Keap1 groups under different conditions; (e) DHE staining was performed to detect ROS levels in the NC+LPS, NC+LPS+OE-SET8, and NC+LPS+OE-SET8+OE-Keap1 groups under different conditions.

We investigated whether SET8 regulates oxidative stress via the KEAP1-Nrf2-ARE signaling pathway and thus affects astrocyte autophagy and glial scar formation. First, we used western blotting to detect SET8, KEAP1, and Nrf2-related proteins in the signaling pathway. Compared with the LPS+OE-SET8+OE-KEAP1 group, the LPS+OE-SET8 group significantly decreased the expression of KEAP1 and promoted SET8 and Nrf2 (Figure 4(a)). Next, we detected ROS levels using DHE staining and found that the LPS+OE-SET8 group had significantly reduced ROS content (Figure 4(e)). Meanwhile, compared with the LPS+OE-SET8+OE-KEAP1 group, LPS+OE-SET8 significantly decreased the expression of astrocyte signature proteins, autophagy-related proteins LC3II/I and Beclin1, and glial scar formation-related proteins according to the western blotting results (Figures 4(b)–4(d)). These results suggest that SET8, through activation of the KEAP1-Nrf2-ARE signaling pathway, inhibits oxidative stress and suppresses astrocyte autophagy as well as glial scar formation, thereby promoting recovery after SCI.

### 3.5. SET8 Promotes Motor Function Recovery after SCI in Rats In Vivo

**Figure 5:**
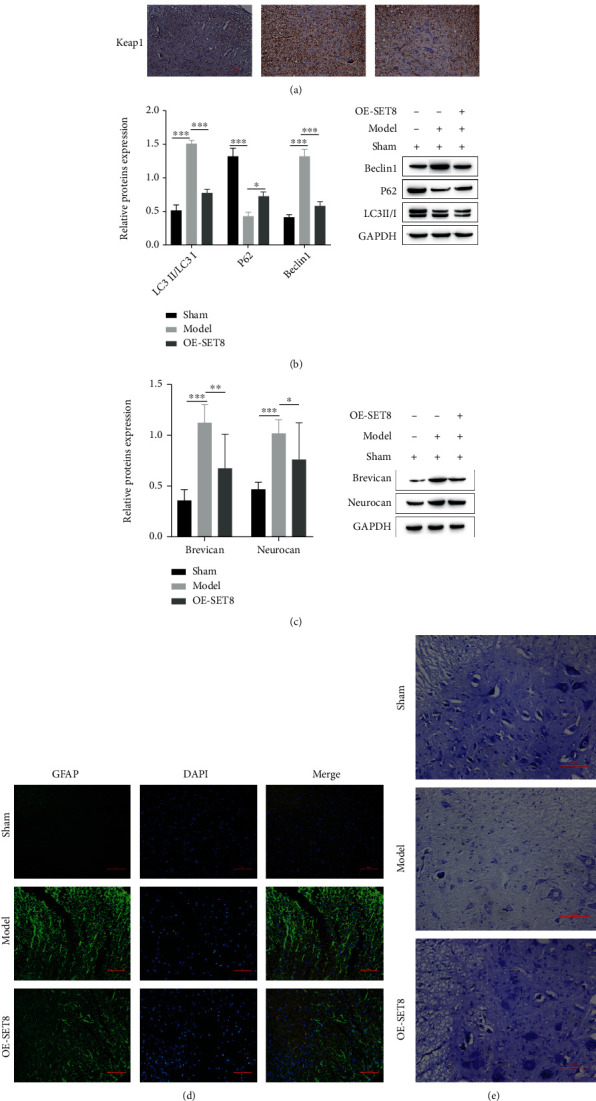
SET8 promotes motor function recovery after SCI in vivo in rats. (a) Analysis of SET8 and Keap1 expression in the sham, model, and OE-SET8 groups by immunohistochemistry; Western blotting of the astrocyte autophagy-related proteins LC3II/I, P62, and Beclin1 in (b), the glial scar formation-related proteins brevican and neurocan in the sham, model, and OE-SET8 groups in (c), and OE-SET8 expression in the different groups. (d) Immunofluorescence analysis of astrocyte morphology and glial scar formation in the sham, model, and OE-SET8 groups. The number of surviving neurons in the sham, model, and OE-SET8 groups was detected by Nissl staining. (e) The number of surviving neurons was detected by Nissl staining in the sham, model, and OE-SET8 groups.

We further validated that SET8 promotes motor function recovery by regulating the KEAP1-Nrf2-ARE signaling pathway after SCI in rats. We simulated post-SCI rats and established an in vivo model. First, we analyzed the expression of the key genes SET8 and KEAP1 after SCI in rats by immunohistochemistry (Figure 5(a)), and the experimental results were similar between the in vivo and in vitro experiments. Next, we detected astrocyte autophagy proteins using western blotting, and the experimental results showed that OE-SET8 significantly decreased the level of astrocyte autophagy, and the autophagy-related proteins LC3II/I and Beclin1 were diminished in astrocytes in OE-SET8-induced tissues, while P62 expression was enhanced (Figure 5(b)). Meanwhile, western blotting assays showed that the glial scar formation-related proteins Brevican and Nucrocan were downregulated (Figure 5(c)). The immunofluorescence assay also showed more visually that in the Model group, a wide thick glial scar was formed by proliferating, hypertrophic astrocytes, while in the OE-SET8 rats, normal, more elongated astrocytes were observed, and the glial scar was shorter and narrower (Figure 5(d)) [32]. Here, we obtained a good phenotypic characterization of the astrocytes observed in the in vivo animal group.

Neurons are known to be critical for recovery after SCI, and Liu et al. [33] showed that neurons innervate changes in neuroplasticity and improve functional recovery after SCI in the spinal cord. However, after SCI, neuronal death occurs in large numbers and is largely driven by reactive astrocytes [34, 35]. The results of this study are presented below. To determine the effect of OE-SET8 on neurons in rat SCI, we used Nissl staining to examine the neurons in different groups. After SCI in rats, the number of surviving neurons decreased in the SCI group, while there was a larger number of surviving neurons in OE-SET8-induced SCI tissue (Figure 5(e)).

In summary, we fully validated in vivo that SET8 can inhibit the autophagy phenomenon in astrocyte and glial scar formation by regulating the KEAP1-Nrf2-ARE signaling pathway to improve neuronal survival after SCI in rats, thereby promoting the recovery of motor function after SCI in rats.

## 4. Discussion

SCI usually results in disability or even paralysis, because the oxidative stress triggers astrocyte activation, glial scar formation, and neuronal cell death in the lesion[36], which severely hinders prominent regeneration and motor function recovery in SCI. Currently, there is no effective clinical treatment for the recovery of motor function after SCI. In this study, we studied the activation of the Keap1-Nrf2-ARE signaling pathway by SET8/H4K20me1 to inhibit oxidative stress-induced autophagy in astrocytes and promote recovery of motor function after SCI.

After traumatic injury to the CNS, including SCI, the surrounding astrocytes are reactive, proliferate, hypertrophy, and migrate to the lesion site, intertwining to form a glial scar [37–39]. This pathological phenomenon leads to the production of axon growth inhibitors and prevents the regeneration of the spinal cord. In the mammalian central nervous system, axon regeneration can be hindered by the formation of glial scars, which is the main reason for the low regeneration ability [40]. In this study, glial scar formation mediated by LPS-induced astrocyte activation after SCI was investigated in rats. LPS-induced astrocyte activation was associated with upregulation of GFAP, LC3II/I and Beclin1, downregulation of P62 and enhanced autophagy. Brevican and versican are produced by reactive astrocytes in glial scars, are the main inhibitory extracellular matrix molecules [41], and play a key role in the regeneration and motor function of the spinal cord in rats. Western blotting results showed that neuroglial scar brevican and versican were significantly elevated after LPS-induced enhancement of astrocyte autophagy. In our vivo experiments, we also foundthat astrocytes sequentially exhibited phenotypically distinct changes, first increasing the number of hypertrophied reactive astrocytes and then developing into glial scars after SCI. It was suggested that the enhanced autophagic capacity of activated astrocytes plays a key role in the recovery of motor function in rats [42]. Therefore, the main therapeutic strategy for glial scarring focuses on the regulation of astrocyte activation as well as autophagy [43].

As a nuclear factor red lineage 2-related factor, Nrf2 regulates cellular defense against oxidative damage through its expression in response to oxidative stress [44]. Nrf2 plays a key role in the recovery of motor function after SCI, as it regulates oxidative stress-related molecules such as ROS, thioredoxin (TXN), and glutathione (GSH) [45]. Nrf2 inhibits oxidative stress and blocks the apoptotic cascade by activating Nrf2 in the damaged spinal cord [46]. The mechanism suggests that Nrf-2 is released from Keap-1 and translocates to the nucleus to bind to the ARE, thereby activating antioxidant defense enzymes and attenuating cellular oxidative stress [47, 48]. Several studies have shown that inhibition of Keap1 activates Nrf2, thereby inhibiting oxidative stress [49, 50]. In this study, we revealed that the Nrf2/ARE signaling pathway can be inhibited by LPS-induced astrocyte activation after SCI in rats. In addition, si-Keap1 ameliorated LPS-mediated oxidative damage, as evidenced by a decrease in ROS content by DHE staining and a decrease in the astrocyte marker proteases GFAP and VimentinD by western blotting. The expression of the autophagy-associated proteins LC3II/I and Beclin1 was downregulated by western blotting, and the expression of P62 was upregulated, while the expression of the glial scar-associated proteins brevican and neurocan was low. Therefore, Keap1 could be used as a therapeutic target for motor function recovery after SCI.

In multicellular organisms, SET8 is the only enzyme that produces histone H4 monomethylation on lysine 20 (H4K20me1) [51]. SET8 plays a key role in the epigenetic regulation of genes in many cellular processes [52]. The mechanism of SET8 regulation in human umbilical vein endothelial cells (HUVECs) with hyperglycemia showed that low expression of SET8 increased the production of cellular ROS, leading to increased oxidative stress, while overexpression of SET8 attenuated oxidative stress damage to endothelial tissue by blocking ROS accumulation [27]. However, the mechanism of SET8 regulation in rat SCI is not clear. In this study, our results showed that overexpression of SET8 reversed the LPS-mediated Keap1/Nrf2/ARE signaling pathway, alleviated ROS accumulation, inhibited LPS-mediated astrocyte autophagy and created glial scars, and thus improved the recovery of motor function after SCI. Furthermore, ChIP assays showed that overexpression of SET8 enriched the downstream target H4K20me1 in the Keap1 promoter region and showed by RT–qPCR and western blotting that overexpression of SET8 downregulated Keap1 and upregulated Nrf2 expression. These data fully demonstrate that overexpression of SET8 can inhibit Keap1 to reverse LPS-induced repression of the Nrf2/ARE signaling pathway.

Nissl staining is a commonly used method for neuronal morphology and cytoarchitecture detection [53]. In this study, to further verify the regulatory mechanism of overexpressed SET8 in rat SCI, we detected the survival of neurons in rat SCI tissues by Nissl staining. In the model rat group, there were significantly fewer neurons involved in regulation by overexpressed SET8 after SCI, which fully indicated in vivo that overexpressed SET8 could increase the recovery of motor function after SCI in rats.

In summary, these results showed that SET8 reduces glial scar generation and inhibits astrocyte autophagy through regulation of Keap1/Nrf2/ARE, thereby improving motor function recovery after SCI. OE-SET8/H4K20me1 inhibits Keap1, and the Nrf2-ARE signaling pathway was activated to inhibit oxidative stress-induced autophagy in astrocytes and promote motor function recovery after SCI. Here, we provide information on how SET8 regulates astrocyte activity by the Keap1/Nrf2/ARE signaling pathway after SCI, thereby generating a permissive environment that promotes motor function recovery after SCI, which can help optimize cellular therapies after SCI and develop therapeutic strategies for secondary SCI.

## Figures and Tables

**Table 1:** Gene primer sequences.

Gene	Primer name	Sequence (5′→3′)
SET8	For	AGCTCCAGGAAGAGCAAAGCCGAG
	Rev	GGCGTCGGTGATCTCGATGAGGT
Keap1	For	ACAGCAGCGTGGAGAGATATGAGC
	Rev	GATACAGTTGTGCAGCACGCAGAC
Nrf2	For	CCCAGCACATCCAGACAGACACCA
	Rev	AGACTGAACTTTCAGCGTGGCTGG
GAPDH	For	AGTACCAGTCTGTTGCTGG
	Rev	TAATAGACCCGGATGTCTGGT

## Data Availability

The data used to support the findings of this study are included within the article.
